# Transforming Growth Factor-β Signaling Pathway in Colorectal Cancer and Its Tumor Microenvironment

**DOI:** 10.3390/ijms20235822

**Published:** 2019-11-20

**Authors:** Yoshiro Itatani, Kenji Kawada, Yoshiharu Sakai

**Affiliations:** Department of Surgery, Graduate School of Medicine, Kyoto University, Kyoto 606-8507, Japan; itatani@kuhp.kyoto-u.ac.jp (Y.I.); ysakai@kuhp.kyoto-u.ac.jp (Y.S.)

**Keywords:** TGF-β signaling, colorectal cancer, SMAD4, tumor microenvironment

## Abstract

Transforming growth factor-beta (TGF-β) signaling is one of the important cellular pathways that play key roles for tissue maintenance. In particular, it is important in the context of inflammation and tumorigenesis by modulating cell growth, differentiation, apoptosis, and homeostasis. TGF-β receptor type 2 (*TGFBR2*) mutations affected by a mismatch repair deficiency causes colorectal cancers (CRCs) with microsatellite instability, which is, however, associated with relatively better survival rates. On the other hand, loss of *SMAD4*, a transcription factor in the TGF-β superfamily signaling, promotes tumor progression. Loss of heterozygosity on chromosome 18 can case SMAD4-deficient CRC, which results in poorer patients’ survival. Such bidirectional phenomenon driven by TGF-β signaling insufficiency reflects the complexity of this signaling pathway in CRC. Moreover, recent understanding of CRC at the molecular level (consensus molecular subtype classification) provides deep insight into the important roles of TGF-β signaling in the tumor microenvironment. Here we focus on the TGF-β signaling in CRC and its interaction with the tumor microenvironment. We summarize the molecular mechanisms of CRC tumorigenesis and progression caused by disruption of TGF-β signaling by cancer epithelial cells and host stromal cells.

## 1. Introduction

Transforming growth factor-beta (TGF-β) signaling pathway plays critical roles in controlling tissue development, proliferation, differentiation, apoptosis, and homeostasis [1]. As such, disruption of this signaling pathway leads to several diseases including cancers. TGF-β signaling regulates many target genes—either positively or negatively—in a context-dependent manner [2]. Although TGF-β signaling inhibits epithelial growth in normal tissues, it promotes tumor cell progression in tissues with advanced cancer [3]. This phenomenon is known as TGFβ paradox. These context-dependent and paradoxical dynamics of the signaling complicate the understanding of the role of TGF-β signaling in cancer biology. As the effect on the colonic epithelial cells, TGF-β signaling exhibits reduction in cell proliferation, along with promotion of differentiation and apoptosis [4]. In addition to its effect on epithelial cells, TGF-β plays protective roles against luminal bacterial antigens by suppressing intestinal immune cells in the stroma and inducing immune tolerance [5,6]. Therefore, disruption of TGF-β signaling in the colon prompts tumor progression not only via epithelial cells transformation but also via tumor-stromal interactions [7,8,9,10,11].

Recent advances in DNA sequencing technology, such as next-generation sequencing and digital polymerase chain reaction (PCR), re-focuses the importance of DNA alteration in cancer cells. Recently, a novel classification for colorectal cancer (CRC) was advocated as consensus molecular subtype (CMS) based on the following molecular features: CMS1 (microsatellite instability (MSI)-immune) as hypermutated, microsatellite unstable, and strong immune activation; CMS2 (canonical) as epithelial, marked WNT and MYC signaling activation; CMS3 (metabolic) as epithelial and evident metabolic dysregulation; and CMS4 (mesenchymal) as prominent TGF-β activation, stromal invasion, and angiogenesis [12]. Most CRC cells with high levels of MSI (MSI-H) accumulate mutations in TGF-β *receptor type 2* (*TGFBR2*) as it carries microsatellite sequences [13,14]. As these findings indicated, disruption of TGF-β signaling plays a pivotal role in CRC pathogenesis in several molecular types of CRC. Moreover, clinical evidence has revealed its strong involvement in patients’ prognosis after curative resection [15]. In this review, we summarize the proposed mechanisms of TGF-β signaling disruption involved in CRC development, progression, and invasion/metastasis.

## 2. TGF-β Signaling Pathway

### 2.1. TGF-β Signaling in Cell Biology

**Figure 1:**
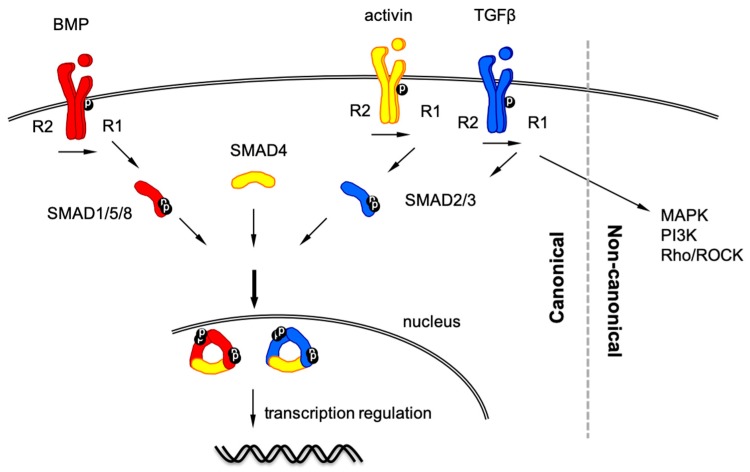
Schematic representation of Transforming growth factor-beta (TGF-β) superfamily signaling pathway.

TGF-β superfamily signaling involves > 30 components, mainly divided into two subfamilies: the TGF-β-activin-nodal subfamily and the bone morphogenetic protein (BMP) subfamily [1,2,16] (Figure 1). TGF-β ligands assemble their corresponding receptors: two type 1 components and two type 2 components. Type 2 receptors serve as activators to phosphorylate type I receptors, whereas type 1 receptors function as propagators to transduce the signal downstream to cytoplasmic proteins. Components of both receptors are serine/threonine kinases. After ligand binding, BMP type 1 receptors phosphorylate SMAD1/5/8 (the abbreviation of SMAD refers to the homologies of SMA (small worm phenotype of *Caenorhabditis elegans*) and *Drosophila* MAD (Mothers Against Decapentaplegic), whereas TGF-β type I receptors and activin type 1 receptors phosphorylate SMAD2/3. These sets of SMAD proteins are known as receptor-regulated SMAD (R-SMAD). Phosphorylation of two C-terminal serine residues on R-SMAD facilitates trimerization with two R-SMAD molecules and one SMAD4 (also known as common-mediator SMAD, Co-SMAD). This SMAD trimer plays a central role in the TGF-β superfamily signaling to translocate to the nucleus and bind DNA via their DNA binding site. The CAGAC motif or its complementary sequence TCTG motif is referred to as the SMAD binding element (SBE) where SMAD2/3/4 mainly bind. In addition, SMAD1/5/8 and SMAD2/3/4 can also bind the GC-rich element when activated [4,17]. R-SMAD molecules in the SMAD4-R-SMAD complex can also bind other transcription factors as partners to regulate their transcription. In addition to the canonical SMAD-dependent pathway, TGF-β superfamily ligands transduce non-canonical, SMAD-independent pathways such as various mitogen-activated protein kinase (MAPK), phosphoinositide 3-kinase (PI3K)/Akt and Rho/Rho-associated protein kinase (ROCK) pathways [18].

Similar to most signaling pathways, TGF-β signaling is regulated from the ligand level to the effector level. Most of the TGF-β ligands act as paracrine fashion, and their access to the cognate receptors is regulated by ligand-binding proteins such as soluble proteins and extracellular matrix [19,20]. In addition to the signal-transducing SMAD members (R-SMAD and Co-SMAD), another type of SMAD inhibits TGF-β signaling pathway (inhibitory SMAD, I-SMAD). SMAD6/7 (I-SMAD) inhibits signal transduction by interfering with the phosphorylation of R-SMAD from type 1 receptors. Ubiquitination of R-SMAD/Co-SMAD is also one mechanism for signal degradation [21].

### 2.2. TGF-β Signaling in Clinical Situation of CRC Patients

Molecular classification of CRC can provide biological interpretability of CRC. In CMS1 CRC, MSI-H provides accumulation of many somatic gene mutations including *TGFBR2*, which results in the release of tumor neoantigens and activation of tumor immunity. It was reported that MSI-H was an independent favorable prognostic factor for Stage II/III (no distant metastasis) CRC patients after curative resection [15]. In fact, CMS1 CRC exhibits a low metastatic rate compared with other subtypes [22]. Although *TGFBR2* mutation itself is not a favorable prognostic factor within MSI-H CRC population, MSI-H CRC patients exhibit better prognosis compared to microsatellite-stable ones [15,23]. However, once metastasized, CMS1 CRC exhibits poorer survival. One of the reasons of poor prognosis after metastases would be that MSI-H confers resistance to 5-fluorouracil (5-FU)-based chemotherapy [24].

MSI-H derived from deficient mismatch repair (dMMR) accounts for only 15% of CRCs. Alternatively, approximately 85% of invasive CRCs exhibit chromosomal instability (CIN) and loss of heterozygosity (LOH) in some chromatin areas [25]. CIN CRCs accumulate driver mutations such as *adenomatous polyposis coli* (*APC*), *TP53*, *SMAD4*, *KRAS*, and *PI3K catalytic subunit-α* (*PIK3CA*), which Vogelstein and colleagues advocated as the adenoma-carcinoma sequence [26]. In this sequence, TGF-β signaling pathway also plays a pivotal role in CRC progression. In fact, absent expression of SMAD4 (a key transcription factor for TGF-β signaling) is an independent poor prognostic factor after curative surgery for Stage II/III CRC and CRC liver metastases [15,27,28,29]. Moreover, CMS4 carrying the mesenchymal phenotype with TGF-β-activated stroma showed the worst prognosis with low benefit from chemotherapy among all molecular classes [12,30,31,32]. In this scenario, TGF-β signaling causes epithelial-to-mesenchymal transition (EMT) in cancer cells, resulting in an aggressive phenotype [18]. To this end, TGF-β signaling directs serrated adenomas to the mesenchymal CRC subtype, while *TGFBR2* mutation impairs EMT [33,34]. Collectively, these basic and clinical data indicate that disruption of TGF-β signaling, especially in advanced CRC, results in an aggressive phenotype of CRC and, consequently, poor prognosis.

## 3. TGF-β Signaling in Cancer Cells

### 3.1. TGFBR2 Mutation in Cancer Cells

For appropriate function of TGF-β signaling, active TGF-β receptors (both type 1 and 2 receptors) are mandatory [19]. *TGFBR2* mutations are frequently found in MSI-H CRC [35]. MSI-H CRC cells carrying dMMR harbor silent expression of mismatch repair genes through either germline mutations of MMR genes such as *MutL homolog 1 (MLH1)*, *MutS homolog 2 (MSH2)*, *MSH6*, and *Postmeiotic segregation increased 2 (PMS2)*, or *MLH1* promoter hypermethylation [36,37]. Lynch syndrome is an autosomal dominant hereditary cancer syndrome that carries germline mutations of one of these 4 MMR genes, resulting in the development of many types of cancers including CRC, endometrial, ovarian, gastric, small intestine, pancreatic, and urothelial tract cancers [38]. Lynch syndrome accounts for approximately 3% of CRCs, whereas approximately 12–15% of CRCs is sporadic MSI-H CRC, resulting from hypermethylation of the *MLH1* promoter [39]. Because *TGFBR2*, carrying a 10-adenine repeat, is only one of the genes affected by dMMR, it is possible that *TGFBR2* mutation is merely a bystander event [13,40]. However, according to several mouse studies, *TGFBR2* mutation itself does have the potential to transform normal colonic epithelial cells to malignant cells [41,42]. In addition to *TGFBR2* mutation, *BMP receptor type 2 (BMPR2)* can be also affected by dMMR as it also carries a 7-adenine repeat that is also affected by dMMR [43].

**Figure 2:**
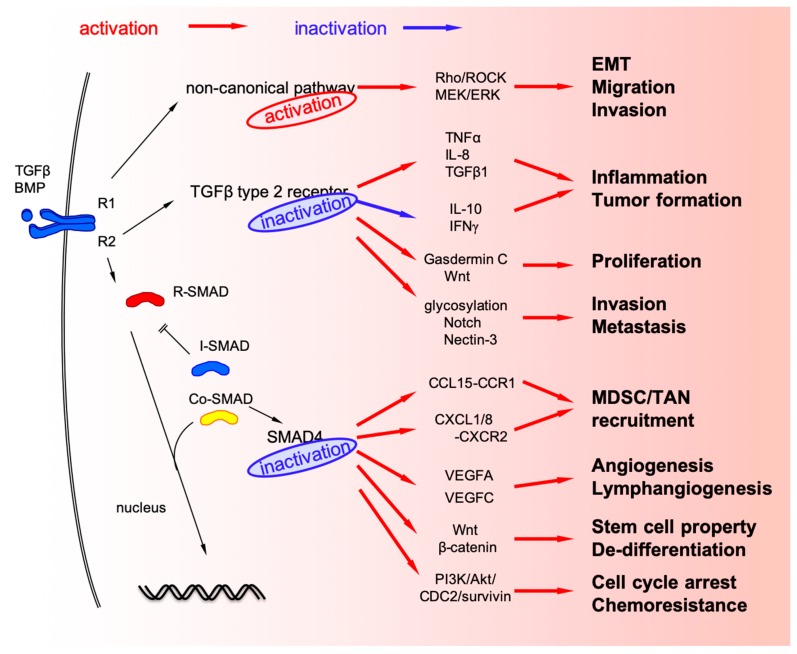
TGF-β alteration in colorectal cancer (CRC) cells.

MSI-H CRC exhibits high immune response that can be targeted for immune checkpoint inhibitors [44]. Although high immune response in MSI-H CRC is the result of tumor neoantigen load caused by hypermutation, TGFBR2 impairment can also directly promote inflammation in the tumor microenvironment of CRC. *TGFBR2* deficiency in an *APC*-deleted mouse model of intestinal adenoma increased inflammatory burden and promoted tumor progression via producing tumor necrosis factor-α (TNF-α), interleukin (IL)-8, and TGF-β1 as well as suppressing of anti-inflammatory cytokines such as IL-10 and interferon (IFN)-γ, which resulted in increased infiltration of CD11b^+^Gr1^+^ granulocyte population into the tumor microenvironment (Figure 2) [45]. Moreover, TGFBR2 disruption in combination with inflammation in the colon causes invasive CRC via tumor-associated macrophage (TAM) infiltration [46].

TGFBR2 inactivation in CRC cells contributes to the malignant phenotype via multiple pathways such as Wnt-β-catenin, Hippo, and MAPK [47]. In a mouse model designed to conditionally knockdown *TGFBR2* in the proximal colon, TGFBR2 impairment in combination with Wnt-β-catenin pathway activation promoted the upregulation of Gasdermin C, which stimulated the proliferation of CRC cells [48].

Glycosylation is an important post-translational protein modification and also affects malignant phenotype of cancer cells [49]. TGF-β signaling can also modulate the protein glycosylation pattern in MSI-H CRC. TGFBR2 impairment in MSI-H CRC cell line, HCT116, can upregulate some of glycosylation-related genes and affect important cell signaling pathways such as Notch [50]. In this cell line model, Nectin-3 (a cell surface glycoprotein that modulates cancer cell invasion and metastasis) was also upregulated when *TGFBR2* was impaired, and reconstitution of TGFBR2 upregulated growth differentiation factor-15 (GDF15; one of the ligands of TGF-β superfamily signaling) in a cell line model [51]. *TGFBR2* mutation in CRC can also cause changes in the components secreted by cancer cells. For example, in vitro experiments with MSI-H TGFBR2-deficient HCT116 cells revealed that extracellular matrix and nucleosome-related proteins were upregulated, while proteasome-associated proteins in the extracellular vesicles were downregulated [52,53]. Clinical implications of these cell line-based experiments are, however, not fully understood.

### 3.2. Mutations and Deletions of SMAD4 (Co-SMAD) in CRC Cells

LOH is one of the common features of carcinogenesis that causes cancer cells to lose tumor suppressor genes and acquire malignant phenotype [54]. Chromosome 18q21 is frequently affected by LOH in microsatellite-stable CRC. There are many genes in this chromosomal region including *SMAD2*, *SMAD4*, and *deletion of colon cancer* (*DCC*) that may contribute to form malignant phenotype of CRC. Among the genes on chromosome 18q21, *SMAD4* is the established tumor suppressor gene, and loss of SMAD4 disrupts canonical TGF-β signaling because it is a transcription factor for the signaling [54]. Moreover, The Cancer Genome Atlas (TCGA) database revealed that *SMAD4* is one of the most frequently mutated genes in CRC [55].

*SMAD4* is one of the key driver genes that contribute to CRC progression and metastasis [56,57]. Consequently, *SMAD4* loss onto *APC* mutation in intestinal epithelial cells causes malignant invasive phenotype in mouse models [58,59]. Loss of SMAD4 protein expression is found approximately 20–40% of human CRCs [8,9,10,11,60,61]. Although LOH can be the main cause of SMAD4 loss in CRC, there are other proposed mechanisms that contribute to SMAD4 defect in post-transcriptional and post-translational regulation: ubiquitylation, sumoylation, and mircoRNA interference [62,63].

SMAD4 acts as a transcription factor by forming trimers with R-SMAD components and directly regulates target genes. R-SMAD-SMAD4 complexes can also associate with DNA-binding partners to act as a transcription co-factor [4]. Therefore, there are many target genes regulated by SMAD4, indicating that several changes in cancer cells happen when R-SMAD-SMAD4 complexes are disrupted [64,65,66]. RNA sequencing comparing *SMAD4*-proficient and *SMAD4*-deficient colonic epithelial cells in mice revealed upregulation of many inflammation-related genes [66]. Among them, *Ccl9*, one of the C-C motif chemokine ligands, is upregulated in a *SMAD4*-deficient intestinal tumor mouse model [59]. In this mouse model, chemokine expression recruits myeloid cells with corresponding receptors, Ccr1, to promote tumor invasion and metastases [59,67,68]. Similar interactions between SMAD4-deficient CRC cells and surrounding myeloid-derived cells via chemokine signaling was observed in human CRC samples [8,9,10,11]. Namely, loss of SMAD4 from CRC cells promotes upregulation of C-C motif chemokine CCL15 (human orthologue of mouse Ccl9) to recruit CCR1^+^ myeloid-derived suppressor cells (MDSC) [8,9,10,69]. SMAD4-deficient CRC cells also produce C-X-C motif chemokine ligand CXCL1/8 to recruit its corresponding receptor CXCR2^+^ tumor-associated neutrophils (TAN) [11]. MDSC accumulation is characteristic of CMS4 immune contexture [70]. These experimental and observational results suggest that SMAD4-deletion in CRC causes both cancer cell phenotype and tumor microenvironment to switch to the more aggressive cancer phenotype.

TGF-β signaling plays a crucial role in angiogenesis in the tumor microenvironment [71,72]. SMAD4 can also regulate the expression of vascular endothelial growth factor (VEGF)-A and VEGF-C, the main angiogenic factors for tumor angiogenesis and lymphangiogenesis. Disruption of SMAD4 promotes upregulation of these angiogenic factors, resulting in promoting angiogenesis and lymphangiogenesis in CRC [73,74]. TGF-β signaling also plays a critical role in the differentiation of epithelial cells. Loss of SMAD4 promotes β-catenin expression, and simultaneous SMAD4 loss and Wnt activation in the intestinal epithelium trigger the acquisition of stem cell properties and lead to de-differentiation and rapid adenoma formation in the differentiated intestinal epithelium of the Cre-driven conditional mouse model [75,76].

Chemoresistance is also an important property of malignant phenotype of cancer cells, and loss of SMAD4 is a predictive biomarker for 5-FU-based chemotherapy [77,78]. SMAD4 deficiency activates PI3K/Akt/cell-division cycle 2 (CDC2)/survivin pathway to attenuate G1/2 cell cycle arrest, providing resistance to 5-FU-based chemotherapy because 5-FU basically acts as a thymidylate synthase inhibitor to block DNA replication [79,80]. 

SMAD4 loss-induced changes in vivo and in vitro were summarized in Table 1 and Table 2. 

### 3.3. Non-Canonical TGF-β Signaling Pathways in CRC

Non-canonical TGF-β signaling pathways also modulate important cellular physiology. Disruption of non-canonical TGF-β pathways as well as canonical ones is also frequently found in CRC. Although loss of SMAD4 may lead to the blockade of canonical TGF-β signaling, it alters BMP signaling via non-canonical pathway to promote CRC metastasis through activation of Rho/ROCK pathway, leading to EMT, migration, and invasion [81]. Loss of SMAD4 also activates alternative MEK/ERK pathways to promote cell mortality, migration, and invasion [82,83].

## 4. TGF-β Signaling in Stromal Cells in the Tumor Microenvironment

**Figure 3:**
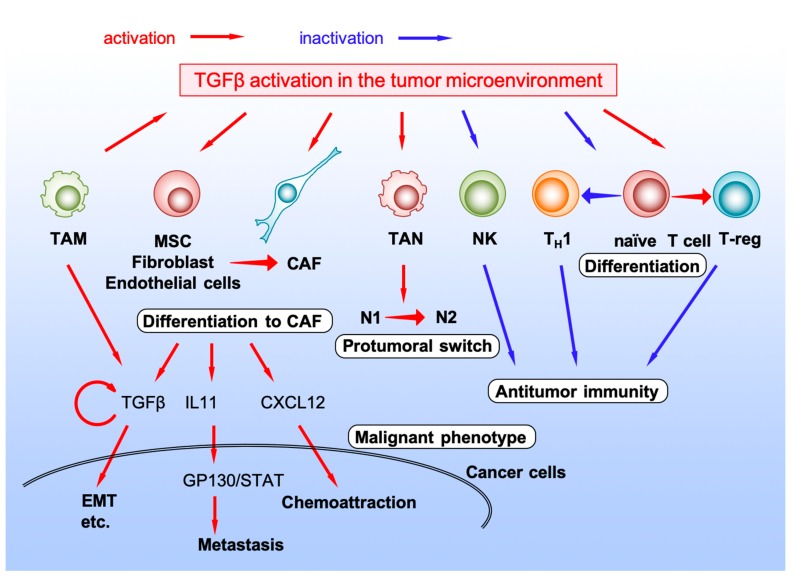
TGF-β activation in the tumor microenvironment.

Cancer tissues contain abundant stromal cells in addition to cancer epithelial cells. Interaction between cancer cells and surrounding stromal cells can either promote or inhibit cancer progression. In the tumor microenvironment, various cytokine/chemokine networks play pivotal roles in controlling the interaction between cancer cells and stromal cells [84,85]. As described above, CMS4 mesenchymal phenotype with poor prognosis characterizes prominent TGF-β activation [12]. These observations suggest that TGF-β activation in the tumor microenvironment can promote the tumor-stromal interaction to induce a malignant CRC phenotype and poorer prognosis (Figure 3).

### 4.1. Cancer-Associated Fibroblast

Cancer-associated fibroblast (CAF) is one of the major components of the tumor microenvironment and act as an important factor in tumor progression and metastasis [86,87,88]. TGF-β activation in the tumor microenvironment promotes differentiation of mesenchymal stem cells (MSCs) to CAFs, activating phosphorylation of signal transducer and activator of transcription 3 (STAT3) and nuclear localization of p-STAT3 via Janus kinase (JAK)/STAT pathway [86]. Normal fibroblasts or endothelial cells can also be converted to CAFs by stimulation of TGF-β superfamily ligands, such as nodal or TGF-β2, to support tumor growth [89,90]. TGF-β ligands also directly activate CAFs; activated CAFs produce chemokine CXCL12 and interact CXCR4^+^ CRC cells, or IL-11 to activate CRC cells through GP130/STAT signaling to metastasize to distant organs [91,92,93]. CAFs secrete TGF-β ligand from themselves to accelerate malignant phenotype of CRC in the hypoxic condition that normally occurs in vivo within the tumor microenvironment [94,95].

### 4.2. Natural Killer Cell

Natural killer (NK) cells can be expanded *ex vivo*, activated and transferred to cancer patients to effectively kill cancer cells. However, highly immunosuppressive tumor microenvironment caused, in part, by excessive TGF-β signaling, may limit the activity of NK cells. Exposure to TGF-β ligand decreases the ability of activated NK cells to kill cancer cells ex vivo, while inhibition of TGF-β signaling in the tumor microenvironment preserves the function of highly activated, in vitro expanded NK cells in CRC in vivo [96].

### 4.3. TANs

Peripheral neutrophil-to-lymphocyte ratio (NLR) in patients with CRC predicts the prognosis after treatment [97,98,99,100]. Although higher neutrophil level could be just bystander in patients with poor prognosis, neutrophils may contribute directly to the tumor progression. In the tumor microenvironment of SMAD4-deficient CRC, TANs are recruited via the chemokine CXCL1/8-CXCR2 axis [11]. Tumor microenvironment contains at least two types of neutrophils: N1 with an anti-tumoral phenotype and N2 with a protumoral phenotype [101,102,103]. TGF-β signaling in the tumor microenvironment regulates phenotypical changes in neutrophils to N2 protumoral ones, while TGF-β blockade with a small molecule inhibitor results in the recruitment and activation of N1 antitumoral TANs [101].

### 4.4. TAMs

TAMs in the tumor microenvironment were known to release pro-inflammatory cytokines there to form inflamed environment, which engages in bidirectional interaction between cancer cells and host immune cells [104]. SMAD4 deletion in CRC cells decreased the number of S100A8^+^ monocytes or CD68^+^ TAMs in the tumor microenvironment; this is associated with unfavorable prognoses in CRC patients [105,106]. Recruited TAM in the CRC microenvironment produces TGF-β ligand to promote proliferation and invasion via EMT or VEGF [107,108]. In compound mutant mice that have mutations in *Apc* and *Tgfbr2* in the intestinal epithelial cells, TAM infiltrates into the invasive tumors express membrane-type1-matrix metalloproteinase (MMP), causing MMP2 activation [46]. Although TAMs can be one of the sources of TGF-β ligand expression, the effects of TGF-β signaling on TAMs in CRC is not fully understood [108].

### 4.5. T Lymphocyte

As NLR can predict CRC patients’ prognosis, low levels of T cell infiltration or low activity of type 1 T-helper cells (T_H_1) can also predict poor outcome of CRC patients [109]. Infiltrating T cells are activated through both signals from major histocompatibility complex (MHC)-presented immunogenic peptide antigens to the T cell receptor (TCR) and signals from CD80/86 on the antigen-presenting cells (APC) to T cell surface receptor CD28. However, once activated, T cell expressed co-inhibitory receptors, such as cytotoxic T lymphocyte antigen 4 (CTLA4) and programmed cell death 1 (PD1), and become exhausted T cell [110]. Abundant TGF-β in the tumor microenvironment promotes T cell exclusion and prevents them to acquire T_H_1-effector phenotype [95]. Inhibition of TGF-β using a small molecule inhibitor, galunisertib, unleashed a potent and enduring cytotoxic T cell response against CRC cells and rendered tumors susceptible to anti-PD-L1-PD-1 therapy.

Regulatory T cells (T-regs) are a subpopulation of T cells that modulate immune system and self-antigen tolerance. T-regs express biomarkers such as CD4, forkhead box P3 (FOXP3), and CD25, and differentiates from naïve CD4^+^ T cells by TGF-β stimulation [111]. In the tumor microenvironment, T-regs play a critical role in suppressing tumor immunity and promoting cancer progression [112]. Therefore, enriched TGF-β in the tumor microenvironment of CRC promotes phenotypical changes of T cells to T-regs and tumor progression [113]. A meta-analysis comparing T-reg infiltration and patients’ prognosis reported that high FOXP3^+^ T-reg density was associated with poor overall and disease-free survival in patients with all types of cancer [114]. However, the prognostic role of T-reg infiltration is tumor-dependent, and, among CRC patients, a higher number of tumor-infiltrating FOXP3^+^ T-regs predicts favorable outcome of CRC patients [114,115].

## 5. Discussion

CRC is the second to third most common cause of cancer-related deaths worldwide [116,117]. Recent developments in surgical technology (e.g., laparoscopic or robotic surgery) and novel therapeutic compounds (small molecule inhibitors and molecular targeted antibodies) are promising, and the mortality rate of CRC is gradually decreasing in some western countries [116,117]. However, once metastasized, treatment strategies can be limited, and, if unresectable, outcomes in CRC patients are unfavorable. TGF-β signaling may contribute to this unfavorable outcome in CMS4 and metastasized CMS1 subpopulations. Therefore, comprehensive understanding of TGF-β signaling in both tumor cells and the tumor microenvironment is mandatory for the construction of novel therapeutic strategies.

As discussed above, the disruption of TGF-β signaling in CRC cells generally promotes tumor formation in the early stage, while its activation may promote cancer invasion and metastasis. Moreover, its activation in the tumor microenvironment generally suppresses tumor immunity and supports cancer cell survival. The bidirectional function of TGF-β signaling within cancer cells and multi-directional functions between cancer cells and their microenvironment make effective drug discovery for CRC treatment difficult. This is because simple blockade of TGF-β signaling can activate tumor immunity in the tumor microenvironment but may alter cancer cell phenotypes to more aggressive ones.

## Figures and Tables

**Table 1:** SMAD4 loss-induced changes in vivo (animal models).

Models	Factors Changed	Function Observed
GEMM ^1^ [59,67]	Ccl9 upregulation	CCR1^+^ myeloid cell recruitment
Xenograft [8,9,10]	CCL15 upregulation	CCR1^+^ TAN/MDSC recruitment
GEMM [75]	Wnt activation	Dedifferentiation Stem cell characteristics
Allograft [80]	PI3K/Akt/CDC2/survivin activation	5-FU resistance

^1^ GEMM, genetically-engineered mouse model.

**Table 2:** SMAD4 loss-induced changes in vitro.

Models	Factors Changed	Function Observed
CRC cell lines [11]	CXCL1/8 upregulation	CXCR2^+^ TAN recruitment
CRC cell lines [74]	VEGF-A upregulation	Angiogenesis
CRC cell lines [74]	VEGF-C upregulation	Lymphangiogenesis

## Abbreviations

5-FU	5-fluorouracil
APC	Adenomatous Polyposis Coli
APC	antigen-presenting cell
BMP	bone morphogenetic protein
BMPR2	BMP receptor type 2
CAF	cancer-associated fibroblast
CCL	C-C motif chemokine ligand
CDC	cell-division cycle
CMS	consensus molecular subtype
Co-SMAD	common-mediator SMAD
CRC	colorectal cancer
CTLA4	cytotoxic T lymphocyte antigen 4
CXCL	C-X-C motif chemokine ligand
dMMR	mismatch repair deficiency
EMT	epithelial to mesenchymal transition
FOXP3	forkhead box P3
GDF15	growth differentiation factor-15
GEMM	genetically-engineered mouse model
IL	interleukin
INFγ	interferon-γ
I-SMAD	inhibitory SMAD
JAK	Janus kinase
LOH	loss of heterozygosity
MAPK	mitogen-activated protein kinase
MDSC	myeloid-derived suppressor cells
MHC	major histocompatibility complex
MLH	MutL homolog
MMP	matrix metalloproteinase
MSC	mesenchymal stem cell
MSH	MutS homolog
MSI	microsatellite instability
MSI-H	high level of MSI
PCR	polymerase chain reaction
PD1	programmed cell death 1
PI3K	phosphoinositide 3-kinase
PIK3CA	PI3K catalytic subunit-α
PMS	Postmeiotic segregation increased
R-SMAD	receptor-regulated SMAD
ROCK	Rho-associated protein kinase
SMAD	*Caenorhabditis elegans* SMA and *Drosophila* MAD
STAT	signal transducer and activator of transcription
TAM	tumor-associated macrophages
TAN	tumor-associated neutrophils
TCR	T-cell receptor
TGFβ	Transforming growth factor-beta
TGFBR2	TGFβ receptor type 2
T_H_1	type 1 T-helper cell
TNFα	tumor necrosis factor-α
T-reg	regulatory T-cell
VEGF	vascular endothelial growth factor
